# Circulating epithelial cell counts for monitoring the therapeutic outcome of patients with papillary thyroid carcinoma

**DOI:** 10.18632/oncotarget.20512

**Published:** 2017-08-24

**Authors:** Ching-Ping Tseng, Kong-Kit Leong, Miaw-Jene Liou, Hsueh-Ling Hsu, Hung-Chih Lin, Yi-An Chen, Jen-Der Lin

**Affiliations:** ^1^ Department of Medical Biotechnology and Laboratory Science, College of Medicine, Chang Gung University, Taoyuan, Taiwan, ROC; ^2^ Graduate Institute of Biomedical Science, College of Medicine, Chang Gung University, Taoyuan, Taiwan, ROC; ^3^ Molecular Medicine Research Center, Chang Gung University, Taoyuan, Taiwan, ROC; ^4^ Department of Laboratory Medicine, Chang Gung Memorial Hospital, Taoyuan, Taiwan, ROC; ^5^ Division of Endocrinology and Metabolism, Department of Internal Medicine, Chang Gung Memorial Hospital, Taoyuan, Taiwan, ROC

**Keywords:** circulating epithelial cells, epithelial cell adhesion molecule, papillary thyroid carcinoma, podoplanin, therapeutic response

## Abstract

Loco-regional recurrence or distant metastasis usually leads to the death of patients with papillary thyroid carcinoma (PTC). Whether or not circulating epithelial cells (CECs) count is a valuable marker in monitoring the therapeutic outcome of PTC was investigated. Patients with PTC (n=129) were treated in our medical center and were categorized into 4 groups with excellent (n=45), biochemical incomplete (n=15), indeterminate (n=37), and structural incomplete (n=32) responses. CECs were enriched from the peripheral blood by the PowerMag negative selection system. Three subtypes of CECs expressing epithelial cell adhesion molecule (EpCAM), thyroid-stimulating hormone receptor (TSHR, a marker for thyroid cells), and podoplanin (PDPN, a marker related to poor prognosis in patients with PTC) were defined by immunofluorescence staining, respectively. The median number of CECs (cells/mL of blood) expressing EpCAM, TSHR, and PDPN was 23 (interquartile range 10-61), 19 (interquartile range 8-50), and 8 (interquartile range 3-22), respectively, for patients enrolled in this study. The number of EpCAM^+^-CECs, TSHR^+^-CECs, and PDPN^+^-CECs was statistically different among patients in different treatment response groups without interference from anti-thyroglobulin antibody (P<0.0001). Patients with structural incomplete response had higher counts for all three CECs subtypes when compared to other patients. EpCAM^+^-CECs was better in distinguishing patients with excellent response from structural incomplete response among the three subtypes of CECs. The sensitivity and specificity of the assay was 84.4% and 95.6%, respectively, when the cut off value was 39 EpCAM^+^-CECs/mL. CECs testing can supplement the current standard methods for monitoring the therapeutic outcome of PTC.

## INTRODUCTION

Thyroid cancer is the most common endocrine malignancy and is the fourth and the fifth most common cancer in women worldwide and in Taiwan, respectively [1–3]. The overall incidence of thyroid cancer increased 3% annually from 1974 to 2013 among patients in the United States diagnosed with thyroid cancer [4]. Papillary thyroid carcinoma (PTC) accounts for more than 80% of cases in all subtypes of thyroid cancer [5, 6]. Although patients with PTC usually have a favorable prognosis, recurrence during the first year after initial thyroidectomy is often a poor prognostic indicator and is a challenge in patient management [7]. About 20-30% of PTC patients eventually develop loco-regional recurrence or distant metastasis, which contributes to their death [7, 8].

Patients with macroscopic invasion of tumor into the perithyroidal soft tissues (gross extrathyroidal extension), incomplete tumor resection, distant metastases, postoperative serum thyroglobulin (Tg) suggestive of distant metastases, pathologic N1 with any metastatic lymph node 3 cm in largest dimension, or follicular thyroid cancer with extensive vascular invasion (> 4 foci of vascular invasion) were defined as high risk group in thyroid cancer [8]. According to current guidelines, patients in the high risk group of PTC should have a total thyroidectomy and should be considered to have radioactive iodine (RAI) remnant ablation to eliminate thyroid cancer cells [8]. The therapeutic response has significant implications in the clinical management of PTC. Routine surveillance of the disease status by serum Tg, anti-Tg antibody (anti-TgAb) and medical imaging such as ultrasonography, positron emission tomography (PET), computed tomography (CT), PET-CT, magnetic resonance imaging (MRI), and ^131^I-whole body scintigraphy (^131^I-WBS) is required during treatment [8–10]. Postoperative patients are reclassified into four response to therapy groups which include excellent response, biochemical incomplete response, indeterminate response, and structural incomplete response based on the outcome of medical imaging and the serum level of Tg [8]. A structural incomplete response may lead to additional treatment or ongoing observation depending on multiple clinico-pathological factors such as the tumor size, location, rate of growth, RAI avidity, F-18-fluorodeoxyglucose avidity, and the specific pathology of the structural lesions. About 50-85% of the patients with a structural incomplete response continue to have persistent disease after multiple postoperative RAI therapy. Disease specific death rates could be as high as 11% with loco-regional metastases and 50% with structural distant metastases [8]. Because elevated levels of anti-TgAb in the bloodstream usually interferes with the interpretation of serum Tg testing [11, 12] and medical imaging studies are performed at an interval of 6-12 months, these methods have limitations in characterizing a patient's response to therapy in real time. Additional modalities to monitor treatment outcome remain to be explored.

Liquid biopsies including circulating epithelial cells (CECs)/circulating tumor cells (CTCs), exosomes, cell-free tumor DNA have been demonstrated as feasible biological resources in monitoring treatment response and disease progression for a number of cancers [13–18]. These biological materials are particularly valuable during follow-up of patients whose primary tumor mass has been removed previously by surgery. The prognostic value of CECs expressing different types of surface or intracellular proteins such as the epithelial cell surface marker epithelial cell adhesion molecule (EpCAM), the lymphatic endothelial cell maker podoplanin (PDPN) and the epithelial-mesenchymal transition (EMT) marker vimentin has been found in head and neck cancer, colorectal cancer, breast cancer, prostate cancer and lung cancer [19–26]. The clinical value of liquid biopsies in monitoring the disease status of patients with PTC has only been addressed in a few studies [27–32]. The number of CECs expressing EpCAM or thyroid-stimulating hormone receptor (TSHR) clearly distinguishes cancer-free individuals from patients with PTC, and differentiates patients with PTC as disease-free or with distant metastasis [27]. CECs enumeration further facilitates the detection of distant metastasis for patients who have high levels of anti-TgAb [27]. A case of early stage PTC has been diagnosed based on an unexpected elevated level of EpCAM^+^-CEC in the peripheral blood of a healthy volunteer in a study of thyroid cancer CECs [28]. Whether or not enumeration of CECs expressing EpCAM, TSHR and PDPN provides clinical value in monitoring the response to therapy for PTC patients was explored in this study. The significance of these findings in the clinical management of patients with PTC is discussed.

## RESULTS

### Basic characteristics of the enrolled cases

A total of 129 PTC patients were enrolled in the study between April 2013 and April 2016 to investigate the clinical value of CECs enumeration in the management of thyroid cancer. Forty-one of these patients have been enrolled in our previous study for distinguishing disease status of patients with PTC by CECs [27]. The clinical features of these patients including gender, age at diagnosis, multifocality, thyroid operative method, TNM stage, follow-up duration, the cumulative RAI dose received, the presence of other diseases such as diabetes mellitus and a 2^nd^ primary cancer, and disease free status are summarized in Table 1. Postoperative patients were categorized into groups A, B, C, and D at 6-12 months after determination of serum level of Tg and imaging studies including single or multiple RAI therapy. The clinical features for patients in different response to therapy groups are summarized in Table 2. Group A included 45 PTC patients (10 males and 35 females) who had an excellent response to the treatment with no clinical, biochemical or structural evidence of disease after initial therapy. The median age and the median follow-up duration of these patients was 45 (interquartile range 36-52) years and 8.1 (interquartile range 4.5-13.1) years, respectively. Group B included 15 PTC patients (2 males and 13 females) who had a biochemical incomplete response with abnormal serum Tg (serum Tg > 0.1 ng/ml) in the absence of localizable disease. The median age and the median follow-up duration of these patients was 43 (interquartile range 31-54) years and 5.7 (interquartile range 2.5-9.2) years, respectively. Group C included 37 PTC patients (10 males and 27 females) who had an indeterminate response to the treatment. The biochemical or structural findings could not be classified as either benign or malignant. The median age and the median follow-up duration of these patients was 37 (interquartile range 30-47) years and 5.6 (interquartile range 2.5-15.0) years, respectively. Group D included 32 PTC patients (10 males and 22 females) with a structural incomplete response to the treatment. These patients had persistent or newly identified loco-regional or distant metastases. The median age and the median follow-up duration of these patients was 53 (interquartile range 43-69) years and 6.4 (interquartile range 1.9-11.2) years, respectively. The enrolled cases were gender-matched among the four groups with no statistical difference regarding the multifocality, follow-up duration, the occurrence of diabetes mellitus, and a 2^nd^ primary cancer (Table 2).

**Table 1:** Basic characteristics of enrolled patients with PTC

Clinical characteristic	Parameters^a^
Patient number	129 (100.0)
Gender	
Female	97 (75.2)
Male	32 (24.8)
Age at diagnosis (year)	44 (32-54)
Multifocality	35 (27.1)
Thyroid operative method	
Total thyroidectomy	112 (86.7)
Less than total thyroidectomy	17 (13.3)
TNM (AJCC) stage	
Stage I	75 (58.1)
Stage II	15 (11.6)
Stage III	12 (9.3)
Stage IV	27 (21.0)
Follow-up period (year)^b^	6.7 (3.1-11.9)
Accumulative RAI^c^ dose (mCi)^b^	130 (60-290)
External radiation therapy	4 (3.1)
Concurrent diseases	
Diabetes mellitus	15 (11.6)
2^nd^ primary cancer	5 (3.9)
Response to treatment	
Group A: Excellent response	45 (34.9)
Group B: Biochemical incomplete response	15 (11.6)
Group C: Indeterminate response	37 (28.7)
Group D: Structural incomplete response	32 (24.8)

^a^Data represent the median (interquartile range of 25% and 75%), n (% of the group), or as indicated.

^b^Data represent the mean (range).

^c^RAI, radioactive iodide.

**Table 2:** Clinical features of PTC patients in different responses to treatment groups

Clinical characteristic^a^	Group A	Group B	Group C	Group D	p Value
Patient number	45	15	37	32	-
Gender					
Female	35 (77.8)	13 (86.7)	27 (73.0)	22 (68.8)	0.5660
Male	10 (22.2)	2 (13.3)	10 (27.0)	10 (31.2)	
Age at diagnosis (year)	45 (36-52)	43 (31-54)	37 (30-47)	53 (43-69)	0.0004
Multifocality	10 (22.2)	7 (46.7)	8 (21.6)	10 (31.3)	0.2321
Thyroid operative method					0.9233
Total thyroidectomy	40 (88.9)	13 (86.7)	31 (83.8)	28 (87.5)	
Less than total thyroidectomy	5 (11.1)	2 (13.3)	6 (16.2)	4 (12.5)	
TNM (AJCC) stage					< 0.0001
Stage I	31 (68.9)	9 (60.0)	28 (75.7)	7 (21.8)	
Stage II	7 (15.5)	0 (0.0)	2 (5.4)	6 (18.8)	
Stage III	4 (8.9)	2 (13.3)	3 (8.1)	3 (9.4)	
Stage IV	3 (6.7)	4 (26.7)	4 (10.8)	16 (50.0)	
Follow-up period (year)^b^	8.1 (4.5-13.1)	5.7 (2.5-9.2)	5.6 (2.5-15.0)	6.4 (1.9-11.2)	0.3900
Accumulative RAI dose (mCi)^b^	60 (30-90)	240 (105-1010)	210 (60-313)	290 (120-580)	< 0.001
External radiation therapy	-	-	-	4 (12.5)	-
Other diseases					
Diabetes mellitus	2 (4.4)	3 (30.0)	4 (10.8)	6 (18.8)	0.1800
2^nd^ primary cancer	2 (4.4)	0 (0.0)	2 (5.4)	1 (3.1)	0.8190

^a^Data represent the median (interquartile range of 25% and 75%), n (% of the group) or as indicated.

^b^Data represent the mean (range).

### CEC was detectable for patients at the early and advanced stages of PTC

**Figure 1:**
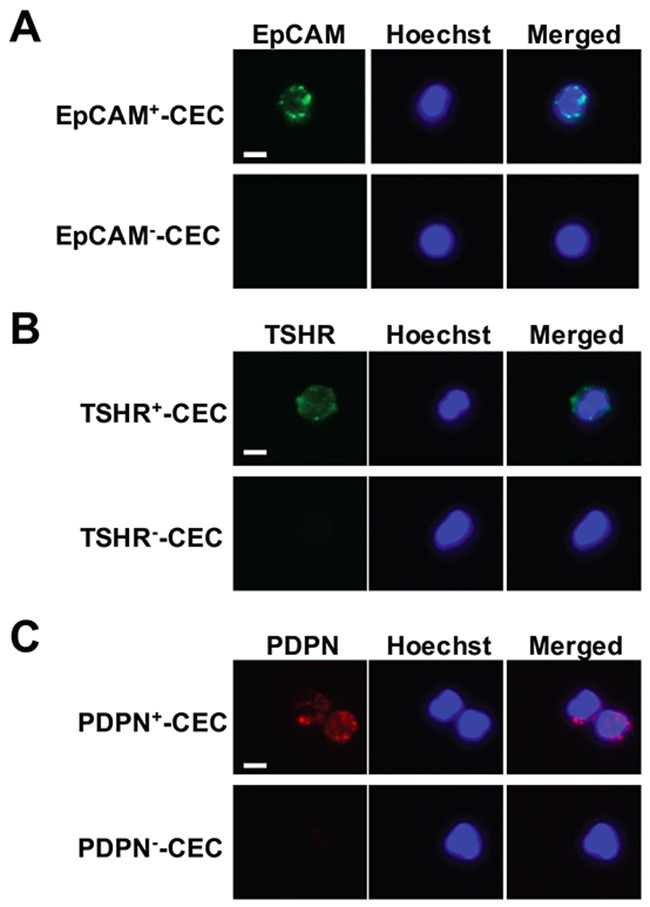
Isolation and characterization of CECs from patients with PTC **(A-C)** CECs were isolated by PowerMag system and analyzed by immunofluorescence staining as described in the Materials and Methods. At least three CECs populations that were positive for EpCAM (panel A, green), TSHR (panel B, green) and PDPN (panel C, red) were defined. Positive staining of Hoechst 33342 (panel A-C, blue) indicates the presence of intact nucleated cells. Bar = 10 μm.

Peripheral blood samples were obtained when patients were in euthyroid status and at 6 weeks after operation or RAI therapy. The treatment course was set by the guidelines of Chang Gung Memorial Hospital. The mean time between initial diagnosis and determination of CEC count was 7.9 ± 5.8 years. Enriched cells were analyzed by immunofluorescence staining using antibodies against EpCAM and TSHR, markers for cells originating from epithelial and thyroid tissue [33, 34], respectively, after RBC lysis and removal of CD45^+^-containing white blood cells from the peripheral blood by the PowerMag system. The number of CECs expressing PDPN, a marker related to a poor prognosis in patients with PTC [35], was also determined. Staining of cells with Hochest 33342 DNA staining dye was used to define nucleated cells. Representative CECs that were positive for EpCAM, TSHR, or PDPN are shown in Figure 1.

The median number of CECs (cells/mL of blood) expressing EpCAM, TSHR, and PDPN was 23 (interquartile range 10-61), 19 (interquartile range 8-50), and 8 (interquartile range 3-22), respectively, in patients (n = 129) enrolled in this study (Table 3). CECs were detected in the peripheral blood from patients at different TNM stages of PTC who had a thyroidectomy and RAI remnant ablation. The number of EpCAM^+^-CECs, TSHR^+^-CECs, and PDPN^+^-CECs was not statistically different between genders. In contrast, the number of EpCAM^+^-CECs (p = 0.0016) and PDPN^+^-CECs (p = 0.0031) but not TSHR^+^-CECs (p = 0.0592) was statistically different among patients at different TNM stages at the time of diagnosis (Table 3). The TSHR^+^-CECs count (p < 0.05) was statistically different between patients at stage I and VI (data not shown). These data indicate that CECs are detectable in patients at both early and advanced stages of PTC and the number of CECs is increased from the early to the late stages of PTC in patients.

**Table 3:** Enumeration of CECs and clinical status of patients with PTC

Parameters	EpCAM^+^-CECs (cells/ml)	TSHR^+^-CECs (cells/ml)	PDPN^+^-CECs (cells/ml)
**Total (n = 129)**	23 (10-61)^a^	19 (8-50)	8 (3-22)
**Gender**			
Female (n = 97)	22 (10-54)	20 (10-44)	8 (3-22)
Male (n = 32)	32 (8-154)	19 (4-72)	9 (2-23)
**P value**	**0.5198**	**0.6526**	**0.9891**
**TNM (AJCC) stage**			
Stage I (n = 75)	17 (8-41)	14 (5-32)	6 (2-13)
Stage II (n = 15)	16 (8-33)	18 (12-34)	9 (3-21)
Stage III (n =12)	28 (17-91)	25 (11-37)	12 (7-22)
Stage IV (n = 27)	58 (28-190)	48 (10-152)	21 (8-40)
**P value**	**0.0016**	**0.0592**	**0.0031**
**Response to treatment group**			
Group A (n = 45)	14 (8-22)^a^	17 (10-26)	6 (3-11)
Group B (n = 15)	27 (5-43)	7 (3-29)	3 (2-20)
Group C (n = 37)	21 (10-57)	12 (5-26)	6 (3-15)
Group D (n = 32)	124 (54-210)	105 (30-235)	34 (18-71)
**P value**	**< 0.0001**	**< 0.0001**	**< 0.0001**
**Anti-TgAb status in Group B and C**			
Anti-TgAb^+^ (n = 18)	21 (12-50)	9 (4-32)	6 (4-13)
Anti-TgAb^-^ (n = 34)	21 (7-54)	12 (4-28)	5 (2-18)
**P value**	**0.8324**	**0.9462**	**0.5061**

^a^The number represents the median (interquartile range of 25% and 75%).

### CECs enumeration for monitoring the response to therapy in patients with PTC

**Figure 2:**
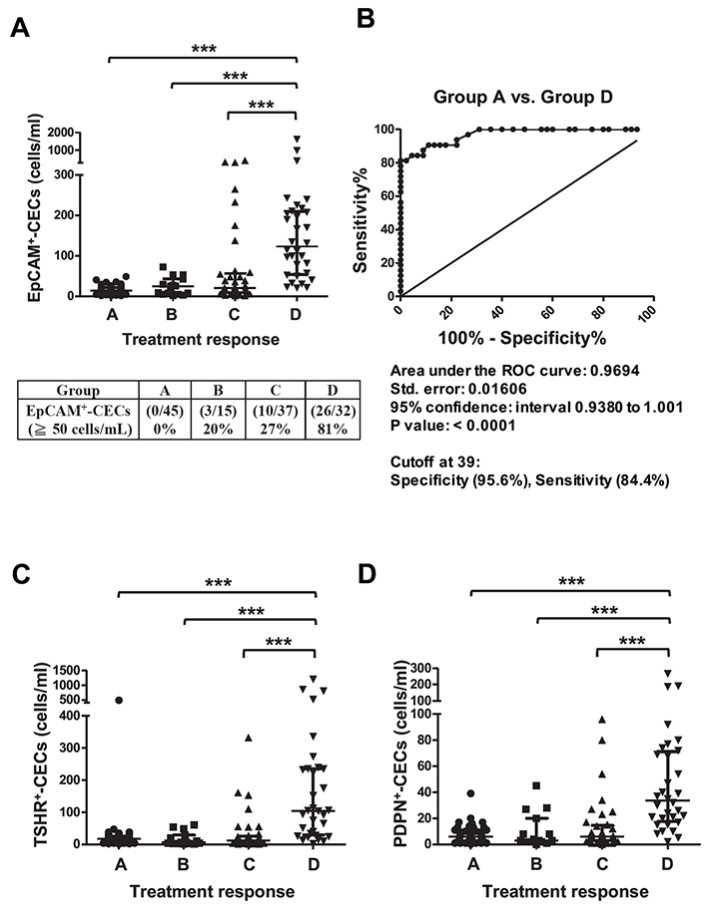
Scatter dot plots and ROC analyses for EpCAM^+^-CECs, TSHR^+^-CECs, and PDPN^+^-CECs counts in different response to therapy groups for patients with PTC **(A, C-D)** The scatter dot plots for the number of EpCAM^+^-CECs (panel A), TSHR^+^-CECs (panel C), PDPN^+^-CECs (panel D) in different response to therapy groups (A, B, C and D). The percentage of patients with the number of EpCAM^+^-CECs ≧50 cells/mL was shown in panel A. Kruskal-Wallis test with the post-hoc Dunn's test were used for statistical analyses. The median and the interquartile range for each group are indicated by the horizontal lines. **(B)** ROC analysis for the number of EpCAM^+^-CECs between group A and group D.

The number of CECs in patients having a different response to therapy was compared and analyzed. The median EpCAM^+^-CECs count was 14 (interquartile range 8-22), 27 (interquartile range 5-43), 21 (interquartile range 10-57), and 124 (interquartile range 54-210) for patients in groups A, B, C, and D, respectively (Table 3 and Figure 2). The number of EpCAM^+^-CECs (p < 0.0001), TSHR^+^-CECs (p < 0.0001), and PDPN^+^-CECs (p < 0.0001) was statistically different among patients having different treatment response categorized in groups A to D. The EpCAM^+^-CECs count was statistically different between groups A and D (p < 0.0001), between groups B and D (p < 0.0001), between groups C and D (p < 0.0001), and among groups A, B, C, and D (p < 0.0001). The number of patients with EpCAM^+^-CECs ≧ 50 cells/mL was 0 (0/45 = 0%), 3 (3/15 = 20%), 10 (10/37 = 27%), and 26 (26/32 = 81%) for groups A, B, C, and D, respectively (Figure 2A). Receiver operating characteristic (ROC) analysis revealed that the EpCAM^+^-CECs count distinguished group A from D with an area under the curve (AUC) equivalent of 0.9694 (p < 0.0001) (Figure 2B). The sensitivity and specificity of the assay was 84.4% and 95.6%, respectively, when the cut off value was 39 EpCAM^+^-CECs/mL. A similar trend was found when TSHR^+^-CECs and PDPN^+^-CECs counts were analyzed and compared among groups A, B, C, and D (Table 3 and Figure 2C and 2D). Most of the patients who were positive for anti-TgAb were in groups B and C. EpCAM^+^-CECs, TSHR^+^-CECs and PDPN^+^-CECs counts were not statistically different when patients in groups B and C, who were anti-TgAb (+) and anti-TgAb (-) were compared (Table 3). These data indicate that anti-TgAb has no effect on CECs counts.

### The clinical value of CECs enumeration in monitoring disease status and management of patients with PTC illustrated by a case of longitudinal follow-up

**Figure 3:**
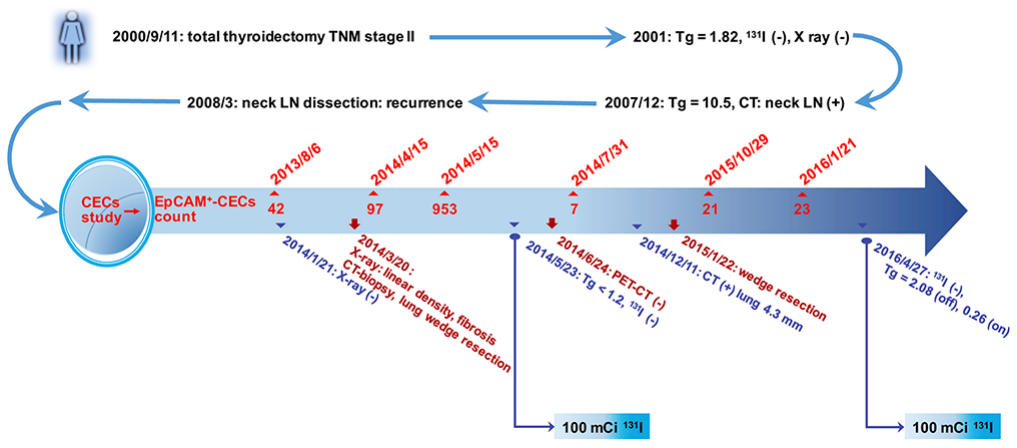
Illustration for the clinical value of CECs counts in the management of a patient with PTC A 63 year-old female PTC patient was enrolled in the study on August 6, 2013. The patient underwent total thyroidectomy on September 11, 2000 with a TNM stage II tumor. The stimulated serum Tg level was < 1.2 ng/mL before enrollment in the study. Due to the elevated CECs count (42 cells/mL), the patient underwent neck and chest CT examination. Following CT-guided biopsy and wedge resection of right upper lung, PTC with lung metastasis was diagnosed histologically. No uptake of ^131^I was observed in the lung in both 100 mCi ^131^I therapeutic scans (2014 and 2016). Final un-stimulated Tg was 0.26 ng/mL.

A female case in this study demonstrates the clinical value of CECs counts in the management of patients with PTC (Figure 3). The patient underwent total thyroidectomy in Sep. 2000 because of the presence of a nodule measuring 2.5 cm in the right lobe of the thyroid. During the operation, neck lymph nodes were not grossly enlarged. No local invasion was exhibited grossly and all macroscopic tumors were resected. The case was defined as low risk at that time and therefore RAI therapy was not performed. After the operation, the patient was treated with 100 μg/day eltroxin. A whole body diagnostic ^131^I scan and a chest X-ray did not show loco-regional or distant metastasis. Un-stimulated levels of Tg fluctuated between undetectable and 1.82 ng/mL in 2001. The un-stimulated level of Tg became gradually elevated to 10.5 ng/mL by the end of 2007. A neck CT scan showed right para-tracheal lymph node enlargement. Selective right lymph node dissection of levels II, III, IV and the lower part of level V was performed on March 8, 2008. The patient had a histological diagnosis of PTC with neck lymph node recurrence. After the second operation, stimulated Tg was undetectable and a 100 mCi ^131^I scan did not show any signs of metastases. The patient status was categorized as in remission. She was enrolled in the current study on Aug. 6, 2013. The EpCAM^+^-CECs count was 42 cell/mL and the unstimulated Tg was 0.25 ng/ml. Further examination show lung metastases, which was proven by CT-guided biopsy and wedge resection of right upper lung in March 2014. An increase in the EpCAM^+^-CECs count was observed after lung wedge resection. The patient had a 100 mCi ^131^I therapeutic scan in May 2014. No metastases were found in Jan 2016 on a follow-up 100 mCi ^131^I therapeutic scan and whole body PET-CT (Figure 3). The EpCAM^+^-CECs count was decreased to 7 cells/mL during the period of treatment. On December 11, 2014, a 4.3 mm lung mass was found by CT scan. The mass was excised by a lung wedge resection. The follow-up EpCAM^+^-CECs count, Tg level, and ^131^I scan all showed no sign of recurrence. This case illustrates that a CECs count is useful in monitoring the disease status and the treatment response of PTC patients.

## DISCUSSION

The prevalence of PTC has increased in many developed and developing countries in recent decades. Although most patients with PTC have an excellent prognosis after appropriate therapy, some patients with loco-regional recurrence or distant metastases have a poor prognosis [7, 36–38]. These patients usually require additional adjuvant treatment to reduce the risk of cancer mortality. In this study, CECs enumeration is shown to supplement the current methods in monitoring the response to therapy for patients at risk of recurrence and distant metastases.

Postoperative stratification of risk for patients with PTC is very important in long term follow-up. Patients with PTC in the high risk group not treated with RAI remnant ablation may have an increased incidence of loco-regional recurrence and distant metastases [8]. Continuous monitoring of the response to therapy is required to provide appropriate clinical care for patients with PTC after thyroidectomy and RAI remnant ablation. Excessive or unnecessary exposure to radio-iodine can affect the patient's quality of life and increase the risk of cellular damage to cause other cancers and diseases [39–41]. In the current clinical setting, medical imaging and serum Tg are the two most important laboratory tests for evaluating the efficacy of a patient's response to therapy [42, 43]. This study demonstrates for the first time that CECs count clearly differentiates those patients who have a structural incomplete response (group D) from those patients with an excellent response, a biochemical incomplete response and an indeterminate response (group A, B, and C). The patients with a structural incomplete response have a significant increase in the number of CECs expressing EpCAM, TSHR or PDPN. Although the absolute CECs count did not differentiate patients in groups A, B, and C, the proportion of patients with a CECs count exceeding a preset threshold (i.e. ≧ 50 cells/mL) was increased from group A to groups B and C. These findings provide a basis for implementing CEC testing as a guideline in risk stratification and in determining whether or not postoperatively aggressive adjuvant therapy is required for patients with PTC. CEC enumeration is also applicable to evaluate the response to therapy for patients with PTC in the high-risk group. At present study, the clinical values of CEC testing were mainly focused on its application as a diagnostic test for clinical outcome. Whether CEC testing provides prognostic value for long term follow-up of PTC-related survival and recurrence is under investigation.

Cancer cells dislodge from the primary tumor mass and enter the bloodstream before metastases are recognized [44, 45]. CECs enumeration in detecting early stage cancer has attracted a lot of attention [46, 47]. A minimal amount of CECs is present in the bloodstream during low tumor burden in the early stages of cancer. Limited sensitivity and specificity are the major challenges for the application of CECs testing in the early detection of cancer [48]. CECs were detected in the peripheral blood of patients with early stage PTC even after thyroidectomy and RAI therapy by the negative selection PowerMag system. The significance of these findings is two-fold: CECs are present in the bloodstream of patients even after therapy; and, the number of CECs in patients with PTC is higher than healthy controls [27]. Hence, monitoring disease status by CECs enumeration is likely beneficial to patients. The ability to detect minimal residual disease after therapy implies that the PowerMag system offers sufficient sensitivity to detect minimal amounts of CECs. This is consistent with the report that CECs isolation by the PowerMag system facilitates the identification of a patient in an early stage PTC [28]. PowerMag is therefore applicable in CECs detection for patients with early PTC and for monitoring minimal residual disease and response to therapy.

Three different proteins including EpCAM, TSHR and PDPN were analyzed by immunofluorescence staining of the CD45^-^ cell filtrate. EpCAM is an epithelial cell surface marker commonly used as the target for detecting either normal or malignant epithelial cells in the bloodstream [33]. TSHR is a marker for the cells originated from the thyroid, although it is also expressed on other cell types [34, 49–51]. With the data of immunofluorescence staining alone, the TSHR^+^ cells are not definitely defined as the cells from thyroid tissue. However, the cohort we analyzed in this study is mainly consisted of patients with PTC. It is logical to consider the TSHR^+^ cells are CECs from thyroid. Both EpCAM and TSHR effectively detect CECs, but these markers do not differentiate normal from malignant cells. The presence of a population of CECs that are positive for PDPN was identified in the current study. PDPN is a lymphatic endothelial marker and among the most frequently up-regulated genes in squamous cell carcinoma, central nervous system tumors and germinal neoplasia [52–54]. PDPN expression is associated with aggressive phenotypes of human cancer and is a poor prognostic marker in thyroid, esophageal, oral, and lung cancers [28, 37, 54, 55]. The metastatic potency of cancer and the frequency of tumor cells embolized in the microvasculature of the lung are correlated with the platelet aggregation activity of PDPN [52, 53]. PDPN mediates the invasive properties of cells derived from PTC and is highly expressed in the neoplastic cells of many papillary thyroid tumors. PDPN was detectable in ~40% of analyzed PTC cases and was absent in the peritumoral margin of “normal” unaffected tissues. PDPN was not detectable in follicular thyroid carcinomas (FTC), follicular adenomas (FA) and normal thyroid tissues [35]. The expression of PDPN is slightly different to the expression of thyroid transcription factor-1 (TTF-1), a marker for thyroid carcinoma. TTF-1 was expressed in normal thyroid follicular cells and parafollicular cells which show diffuse expression pattern [56]. In thyroid cancer, TTF-1 was detectable in 100% of PTC, FTC, and FA; in 90% of poorly differentiated anaplastic carcinomas and medullary carcinomas; and in none to fewer than 25% of undifferentiated carcinomas [56].

Although the three subtypes of CECs are all detectable in the peripheral blood of PTC patients and are able to differentiate the patients with a structural incomplete response from those patients with an excellent response, a biochemical incomplete response and an indeterminate response, EpCAM^+^-CECs enumeration provides greater sensitivity and more specificity when compared to TSHR^+^-CECs and PDPN^+^-CECs. Not all PDPN^+^-CECs and TSHR^+^-CECs are positive for EpCAM, implying that a heterogeneous cell population was present in the CD45^-^ cell filtrates obtained by the PowerMag system. EMT occurs during thyroid cancer progression [57]. Hence, it is likely that some of the PDPN^+^ and TSHR^+^ cells loss the expression of EpCAM and results in the EpCAM^-^PDPN^+^ and EpCAM^-^TSHR^+^ cell populations. These observations are consistent with the observed heterogeneity of CTCs in patients with other cancer types, such as breast cancer, colorectal cancer, and lung cancer [58–60]. It remains to investigate whether or not the PDPN- or TSHR-specific CECs is related to the degree of malignancy and the poor prognosis of patients with PTC.

CECs enumeration is a simple test that is not interfered with anti-TgAb and can be performed more frequently than standard imaging methods. The use of CECs testing for patients with PTC might add information to routine diagnostics. Further studies should aim at investigating the role of CEC as a marker of disease status and its impact on therapeutic decisions.

## MATERIALS AND METHODS

### Study subjects

This study was approved by the Chang Gung Memorial Hospital Institutional Review Board (approval ID: 102-3433B). The enrollment criteria for patients with histologically proven PTC were (1) age ≧ 18 years and (2) ability of the patients to understand and sign the informed consent. Postoperative patients were classified into four groups according to the serum levels of Tg and imaging studies including single or multiple RAI therapy. Group A included the patients with an excellent response to treatment who had no clinical, biochemical or structural evidence of disease after initial therapy (remission, no evidence of disease). This is defined by the negative imaging in neck ultrasonic examination, chest radiography, ^131^I-WBS, CT/MRI/PET-CT and/or bone scan with either suppressed Tg < 0.1 ng/mL when the anti-TgAb was < 115 IU/mL or TSH-stimulated Tg was < 1 ng/mL. Neck ultrasonography studies were conducted on a real-time ultrasonographic machine and a 10 MHz transducer (Aloka). Group B included the patients with a biochemical incomplete response and abnormal Tg values (suppressed serum Tg > 0.1 ng/mL) in the absence of localizable disease. Group C included patients with indeterminate response whose biochemical or structural findings cannot be classified as either benign or malignant (non-stimulated Tg detectable, but < 1 ng/mL). Group D included patients with a structural incomplete response who had persistent or newly identified loco-regional or distant metastases. Distant metastases were defined by a serum Tg > 0.1 ng/mL when the anti-TgAb was < 115 IU/mL and positive medical images in ^131^I-WBS, CT/MRI/PET-CT, chest radiography, or a bone scan.

### Thyroid-related biochemical testing

Anti-TgAb was measured by using a competitive radioimmunoassay (Biocode Hycel, Liege, Belgium) with the analytical sensitivity of 6 IU/mL. Anti-TgAb <115 IU/mL was considered as not interfering with serum Tg testing. Serum Tg was measured by using the highly sensitive Tg Access assay (Beckman Coulter, Brea, CA) when patients underwent levothyroxine (L-T4) treatment to avoid interference from elevated TSH. However, patients may hold L-T4 treatment or injection of recombinant human thyrotropin to monitor the disease status of patients during RAI therapy. The blood samples for CEC testing, serum Tg, and other thyroid-related assays were collected simultaneously from one blood drawing.

### Treatment of patients with PTC

The staging of PTC patients was performed using the International Union Against Cancer Tumor-Node-Metastasis (TNM) criteria (6^th^ edition) [61]. All thyroid cancer tissues were pathologically classified according to the World Health Organization criteria [62]. The American Thyroid Association guidelines [9] were followed for therapeutic planning of the patients. Depending on clinical indications, noninvasive examinations included neck ultrasonography, chest radiography, CT, MRI, bone scintigraphy, PET-CT, and ^131^I-scintigraphy. The patients were subjected to thyroid remnant ablation between four and six weeks after thyroidectomy. The ^131^I ablation dose for most of the patients was 1.1-3.7 GBq (30-100 mCi). WBS was performed one week after ^131^I administration using the dual-head gamma camera (model of Dual head Genesys, Philips/ADAC, Stokesdale, NC; and model of Infinia Hawkeye 4, GE Healthcare, Haifa, Israel) equipped with a high-energy collimator. Subsequently, treatment with L-T_4_ was initiated in order to decrease the level of TSH without inducing clinical thyrotoxicosis. Cases in which the foci of ^131^I uptake extended beyond the thyroid bed were classified as distant metastasis. These patients received higher therapeutic doses of 3.7-7.4 GBq (100-200 mCi). Hospital isolation was arranged for patients requiring dosages exceeding 1.1 GBq. ^131^I-WBS was performed two weeks after administration of ^131^I.

### Enrichment and isolation of CD45^-^ cells

The peripheral blood of the patients was collected for enrichment of CD45^-^ cells by a negative selection system PowerMag [19, 20, 27, 28]. A single enrichment was performed for each patient. Briefly, fresh blood samples (4 ml) were processed by lysis of red blood cells followed by depletion of CD45^+^ white blood cells using a magnetic chamber. The enriched CD45^-^ cells were analyzed by immunofluorescence staining [27].

### Immunofluorescence staining and CECs enumeration

For immunofluorescence staining, leukocyte-depleted cell filtrates were separated into two aliquots. One of the aliquots was incubated with the anti-TSHR antibody (Abcam Inc, Cambridge, England) and the DNA staining dye Hoechst 33342 (Invitrogen Inc, Carlsbad, CA) at room temperature for 1 h. The other aliquot was incubated with the anti-EpCAM antibody (Abcam Inc, Cambridge, England), the anti-PDPN antibody (Angiobio, San Diego, CA) and the DNA staining dye Hoechst 33342 at room temperature for 1 h. After several washes and centrifugation to remove the supernatants, the cell pellets were resuspended and the Alexa Fluor 488-conjugated donkey anti-mouse antibody (for TSHR and EpCAM) and Alexa Fluor 555-conjugated goat anti-rat antibody (for PDPN) were added to the cell suspension. The unbound antibody was removed after incubation in the dark for 30 min. The complete cell aliquot was placed on a slide and the full immunofluorescent images were captured by the fluorescence microscopy using automated slide scanning platform (Zeiss Axiovert 200M) followed by image analysis using the IN Cell Analyzer 1000 Cellular Imaging and Analysis System (GE Healthcare Life Sciences, Pittsburgh, PA). EpCAM^+^-CECs, TSHR^+^-CECs, and PDPN^+^-CECs was defined as the Hoechst-positive cells that were positive for EpCAM, TSHR and PDPN, respectively.

### Statistical analysis

Categorical data were compared using the Pearson chi-squared or Fisher's exact test. CEC counts in different TNM stages and response to therapy groups were compared using the Kruskal-Wallis test for all groups. Dunn's test was used for a post-hoc test between any two groups. ROC analysis was used to illustrate the discrimination ability between any two groups. Statistical analysis was performed using Prism 5 (La Jolla, CA). A p-value < 0.05 was considered statistically significant.

## Abbreviations

Anti-TgAb: anti-thyroglobulin antibody
CECs: circulating epithelial cells
CT: computed tomography
CTCs: circulating tumor cells
EMT: epithelial-mesenchymal transition
EpCAM: epithelial cell adhesion molecule
FA: follicular adenomas
FTC: follicular thyroid carcinomas
MRI: magnetic resonance imaging
PDPN: podoplanin
PET: positron emission tomography
PTC: papillary thyroid carcinoma
RAI: radioactive iodine
ROC: receiver operating characteristic
Tg: thyroglobulin
TNM: tumor-node-metastasis
TSHR: thyroid-stimulating hormone receptor
TTF1: thyroid transcription factor-1
WBS: whole body scintigraphy
